# Cancer therapy's impact on lipid metabolism: Mechanisms and future avenues

**DOI:** 10.3389/fcvm.2022.925816

**Published:** 2022-08-09

**Authors:** Roshni Bhatnagar, Neal M. Dixit, Eric H. Yang, Tamer Sallam

**Affiliations:** ^1^Department of Medicine, David Geffen School of Medicine, University of California, Los Angeles, Los Angeles, CA, United States; ^2^UCLA Cardio-Oncology Program, Division of Cardiology, Department of Medicine, University of California, Los Angeles, Los Angeles, CA, United States; ^3^Division of Cardiology, David Geffen School of Medicine, University of California, Los Angeles, Los Angeles, CA, United States; ^4^Molecular Biology Institute, University of California, Los Angeles, Los Angeles, CA, United States

**Keywords:** lipid metabolism, atherosclerotic disease, chemotherapy, tumor microenvironment, cytotoxic therapy, metabolic dysregulation

## Abstract

Atherosclerotic cardiovascular disease is a growing threat among cancer patients. Not surprisingly, cancer-targeting therapies have been linked to metabolic dysregulation including changes in local and systemic lipid metabolism. Thus, tumor development and cancer therapeutics are intimately linked to cholesterol metabolism and may be a driver of increased cardiovascular morbidity and mortality in this population. Chemotherapeutic agents affect lipid metabolism through diverse mechanisms. In this review, we highlight the mechanistic and clinical evidence linking commonly used cytotoxic therapies with cholesterol metabolism and potential opportunities to limit atherosclerotic risk in this patient population. Better understanding of the link between atherosclerosis, cancer therapy, and cholesterol metabolism may inform optimal lipid therapy for cancer patients and mitigate cardiovascular disease burden.

## Introduction

Dysregulation in lipid signaling is a critical factor for development of atherosclerotic cardiovascular disease (ASCVD). Heightened appreciation for the “causal” association between low density lipoprotein (LDL) levels and ASCVD inspired new paradigms for cardiovascular risk mitigation including stringent emphasis on lipid lowering therapies and a “lower is better” approach for risk reduction (1, 2). However, the influence of dyslipidemia in ASCVD is not uniform across different cohorts, including patients with cancer. In the United States (US), over 17 million patients are survivors of cancer; of which nearly half underwent cytotoxic treatment (3). Cancer biology and therapeutics are strongly linked to changes in cholesterol metabolism and the risk of developing ASCVD (4). The field of “Cardio-Oncology” arose from the need to understand cardiotoxicity of cancer therapeutics, as well as the cross-over mechanisms between cancer biology and cardiovascular physiology (5). Some studies have demonstrated alterations in lipid levels among patients with untreated cancer, suggesting an effect of cancer on lipid homeostasis at a mechanistic level independent of cancer therapeutics, but this area requires further research (6, 7).

**Figure 1:**
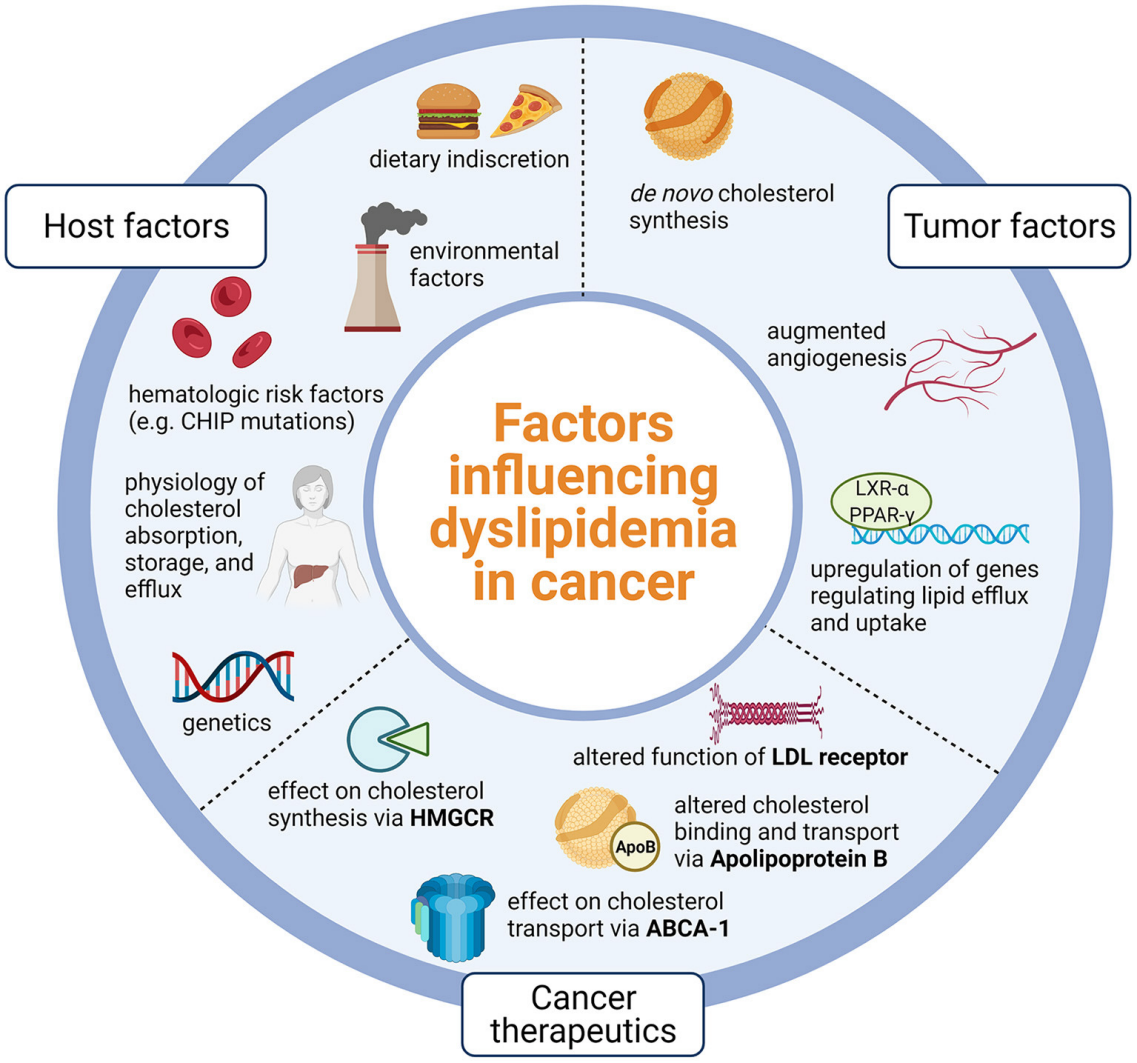
Host factors such as physiology and lifestyle, tumor factors that promote lipid dysregulation, and nuances of cancer therapeutics likely influence dyslipidemia in cancer. Dyslipidemia in cancer is likely due to host physiology and lifestyle, tumor processes that promote lipid dysregulation, and effects of cancer therapeutics on key points of lipid metabolism. CHIP, Clonal hematopoiesis of indeterminate potential; LXR-alpha, Liver X receptor alpha; PPAR-gamma, Peroxisome-proliferator activated receptor gamma; LDL, Low density lipoprotein; HMGCR, 3-hydroxy-3-methylglutaryl coenzyme A reductase; ABCA-1, Adenosine triphosphate binding cassette subfamily A member 1; Created using Biorender.com.

While the cardiotoxic effects of cancer therapeutics, particularly cytotoxic therapies, remain a growing area of research, the direct study of the interweaving of cancer biology, cancer therapeutics, and pathophysiology of ASCVD is less common. Cancer and ASCVD share similar risk factors such as age, tobacco use, and diabetes. However, even after controlling for these risk factors, there is still a strong association between cancer and ASCVD and data indicates that those with a personal history of cancer have 3.42 higher odds of having an elevated ASCVD risk score (8, 9). Likewise, a study of over 6,000 participants of the Multi-Ethnic Study on Atherosclerosis found that those with coronary artery calcium (CAC) score >400 had over 50% increased risk of developing incident cancer over 10 years, even after adjusting for smoking, body mass index, physical activity, hypertension, statin use, and other demographic factors (10). Additionally, recent evidence suggests that hematologic risk factors such as clonal hematopoiesis of indeterminate potential (CHIP) may be linked to accelerated risk of myocardial infarction and poorer prognosis in aortic stenosis and heart failure (11–13). Despite clinical evidence of a link between cancer biology, cancer therapeutics, and the development of atherosclerosis, the exact mechanisms are yet to be elucidated. Moreover, half of cancer survivors do not receive lipid lowering therapy, even though they are eligible (14). In this review, we expand on the interweaving of cytotoxic therapies with cholesterol metabolism, review consequences for increased ASCVD risk in cancer survivors, and suggest strategies for prevention of ASCVD in this high-risk population (Figure 1).

## Overview of lipid biology and atherosclerosis formation

**Figure 2:**
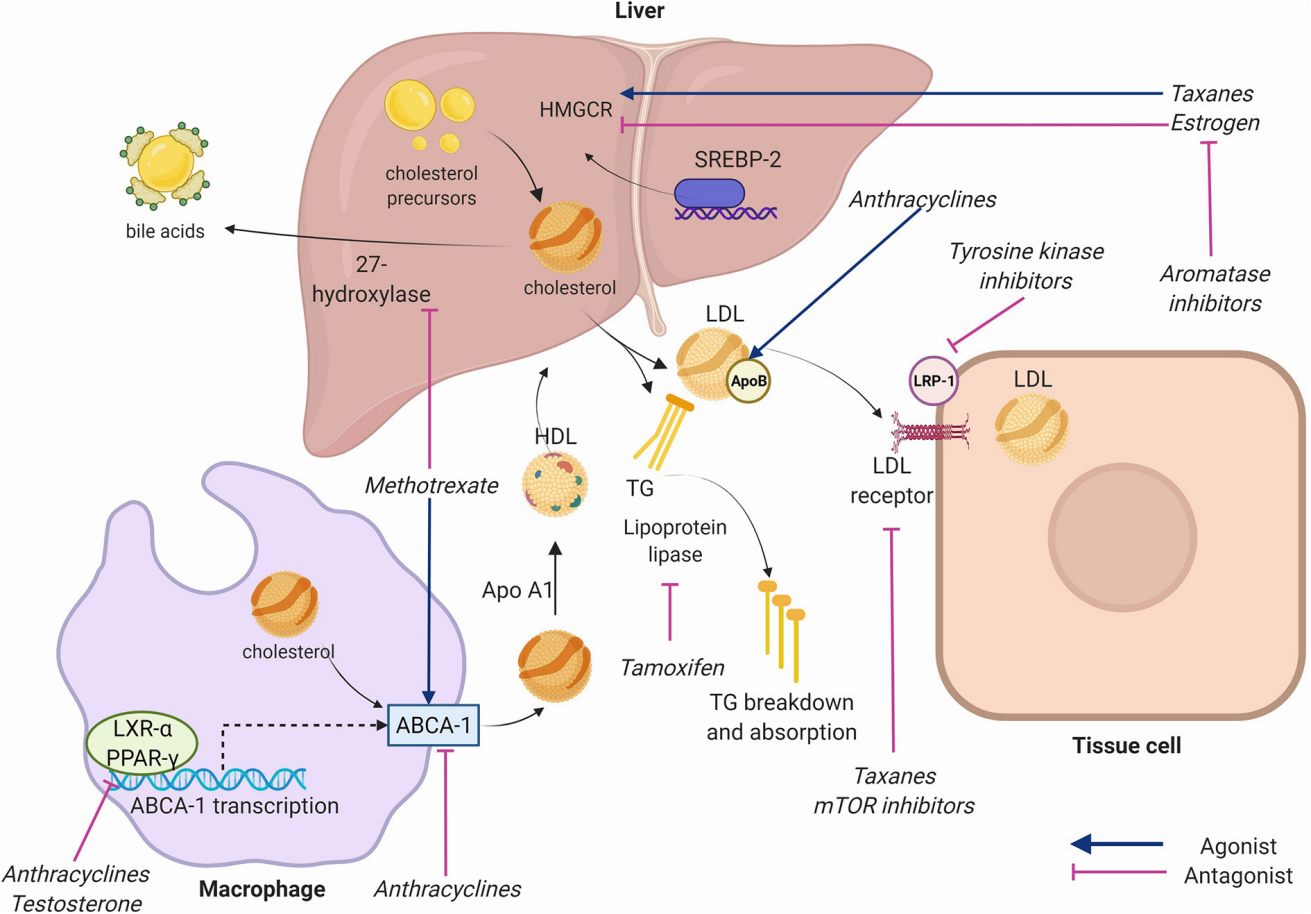
Key receptors and enzymes in cholesterol physiology targeted by chemotherapeutic agents. Anthracyclines inhibit adenosine triphosphate binding cassette subfamily A member 1 (ABCA1) facilitated-transport of cholesterol from cells to high density lipoprotein (HDL), inhibit liver X receptor alpha (LXR-alpha) and peroxisome-proliferator activated receptor gamma (PPAR-gamma) nuclear receptors that transcribe ABCA-1, and increase Apolipoprotein B. Taxanes increase 3-hydroxy-3-methylglutaryl coenzyme A reductase (HMGCR), inhibit Apolipoprotein B, and inhibit low density lipoprotein (LDL) receptor expression. Tyrosine kinase inhibitors (TKIs) inhibit LDL-receptor-related protein (LRP-1). Mammalian target of rapamycin (mTOR) inhibitors inhibit LDL receptors. Methotrexate alters expression of ABCA-1 and 27-hydroxylase. Aromatase inhibitors reduce estrogen function. Estrogen inhibits hepatic HMGCR and reduces cholesterol synthesis. Testosterone inhibits LXR-alpha and PPAR-gamma. Tamoxifen inhibits lipoprotein lipase reducing triglyceride breakdown. Apo A1, Apolipoprotein A1; SREBP-2, sterol regulatory element binding transcription factor 2; TG, Triglycerides. Created using Biorender.com.

Tight regulation of cellular cholesterol stores involves a complex interplay of enzymes that synthesize, absorb, export, and transport cholesterol (Figure 2) (15, 16). Cholesterol is absorbed from the intestine by the intracellular lipid transporter NPC1L1. NPC1L1 is the target of the cholesterol lowering drug ezetimibe. Although the intestine plays a key role in in maintaining cholesterol levels, most cholesterol stores are synthesized *de novo* in the liver. Thus, the liver acts as a master regulator of systemic cholesterol levels and is involved in intricate crosstalk with other metabolically active organs and peripheral tissues to maintain cholesterol homeostasis. Cholesterol biosynthesis requires the coordinated activity of multiple intracellular proteins including the rate limiting enzyme 3-hydroxy-3-methylglutaryl coenzyme A reductase (HMGCR). The liver also regulates the uptake of LDL by regulating the activity of the LDL receptor (LDLR). Both HMGCR and the LDLR are regulated by sterol regulatory element binding transcription factor 2 (SREBP2), which translocates to the nucleus in response to low endoplasmic reticulum cholesterol content to enhance lipid uptake and biogenesis (17). The Liver X receptor (LXR) counterbalances the activity of the SREBPs to maintain cholesterol homeostasis. When cellular lipid stores are high, LXRs are activated and turn on a battery of genes involved in increasing lipid efflux, reversing cholesterol transport and limiting lipid uptake (18). A key gene regulated by LXR is adenosine triphosphate binding cassette subfamily A member 1 (ABCA1), a cell membrane protein that allows for cholesterol efflux from cells. ABCA1 is required for high density lipoprotein (HDL) biogenesis, efflux of cholesterol from macrophages, and reverse transport of cholesterol to the liver (19). Thus, LXR activity within lesions is atheroprotective. Atherosclerosis begins to form as apolipoprotein B lipoproteins accumulate within vessel walls beneath the endothelial lining. This process triggers a maladaptive response that leads to recruitment and proliferation of cells within lesions. Macrophages are key cells within lesions which couple metabolic and immune signaling. The recruitment of monocytes and macrophages and their proliferation within lesions under lipid-rich conditions can lead to lesion progression and many of the hallmark features of advanced plaque including necrotic core buildup, smooth muscle activation, and accumulation of fibrous elements (20). These processes form the basis of major cardiovascular events such as myocardial infarction and stroke (16).

## Cytotoxic therapy and ASCVD risk

A portion of the increased ASCVD risk seen after cancer diagnosis is driven by shared risk factors that predispose to both ASCVD and cancer, such as diabetes mellitus, obesity, and metabolic syndrome (21). However, the cytotoxic therapies involved in cancer treatment can also be a risk factor for ASCVD. In a longitudinal study of 1,413 breast cancer patients with ASCVD, those receiving chemotherapy had 1.7 times higher risk of death due to ASCVD than those not receiving chemotherapy (22). Notably, the difference in morality was not seen until 7 years post-breast cancer diagnosis, likely reflective of the relatively slow progression of ASCVD. Beyond breast cancer, a population cohort study in the United Kingdom found higher risk of coronary artery disease in patients treated with chemotherapy alone for non-Hodgkins lymphoma and a trend toward higher risk in those treated with chemotherapy for lung and breast cancer (23). Immunotherapy and radiation therapy have also been linked to acceleration of atherosclerosis (24, 25). A fundamental issue in this area is the degree to which increased ASCVD risk is attributable to the underlying cancer, cytotoxic therapies, or both. A recent study assessing incident ASCVD among cancer patients found that those with cancer had nearly double the risk of developing ASCVD compared to the population of patients without ASCVD (23.1 vs. 12.0% age-adjusted incidence over 13 years of follow-up). The risk of developing ASCVD was highest among survivors of breast, lung, colorectal, and hematologic cancers, which was independent of traditional cardiovascular risk factors (26). Given the treatability of most cancers, it is unlikely that future studies will evaluate participants with untreated cancer. However, further study of ASCVD risk among cancer patients receiving cytotoxic therapy paired with basic science investigations of these therapies may better elucidate the impact of cytotoxic therapy on ASCVD risk.

As survival among cancer patients improves, the increased risk of death from ASCVD is magnified (27). For example, in breast cancer patients, upwards of 80% of patients are living 5 years after diagnosis (28). Additionally, cytotoxic treatment is becoming a “chronic therapy:” evidence shows duration of chemotherapy and immunotherapy correlates with survival for many cancers (29–31). As cancer survivors live longer and receive more cumulative cytotoxic therapy, they become more likely to die from ASCVD, emphasizing the importance of ASCVD prevention and treatment in this population. Table 1 summarizes the known effects of cancer therapies on cholesterol homeostasis.

**Table 1:** Known effects of cancer therapies on lipid profile and CV risk^a^.

**Class of agent**	**Strength of evidence**	**TC**	**LDL**	**HDL**	**TG**	**Long-term CV risk**
Anthracycline	Moderate	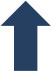	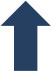	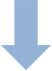	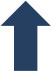	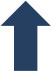
Taxane	Moderate	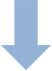	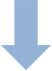	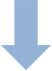	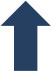	**?**
TKI (Bcr-Abl)	Low	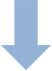	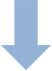	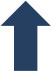	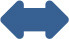	**?**
TKI (VEGF)	Moderate	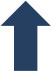	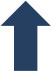	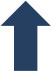	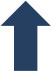	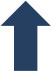
mTOR inhibitor	Low	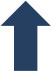	**?**	**?**	**?**	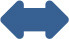
Alkylating agent	Moderate	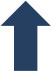	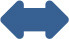	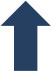	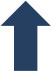	**?**
Platinum	Low	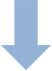	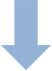	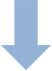	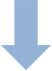	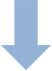
Anti-metabolite	Low	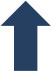	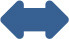	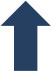	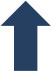	**?**
ADT	Moderate	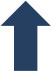	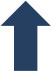	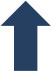	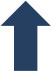	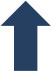
AI	Moderate	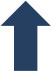	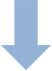	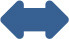	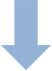	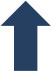
SERM	High	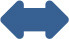	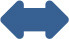	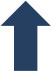	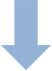	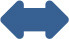
Radiation therapy	Low	**?**	**?**	**?**	**?**	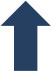
Immunotherapy	Low	**?**	**?**	**?**	**?**	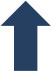
Stem-Cell therapy	Low	**?**	**?**	**?**	**?**	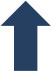

Increased.

Decreased.

Variable effect.

? Unknown.

^a^Please see Supplementary Table 1 for a full set of references used to create this table.

ADT, androgen deprivation therapy; AI, aromatase inhibitor; CV, cardiovascular; HDL, high-density lipoprotein; LDL, low-density lipoprotein, mTOR, mammalian target of rapamycin; SERM, selective estrogen receptor modulator; TC, total cholesterol; TG, triglyceride; TKI, tyrosine kinase inhibitor.

### Effects of chemotherapeutic agents by class on cholesterol homeostasis

#### Anthracyclines

Anthracyclines are among the most widely used chemotherapy agents (32). Their main mechanism of action is *via* topoisomerase II inhibition, mitochondrial impairment, and generation of reactive oxygen species that result in inhibition of DNA and RNA synthesis (33). The most prominent cardiotoxic effect of these drugs is development of cardiomyopathy, however, additional studies have shown a dyslipidemia-inducing effect of anthracycline therapy which may predispose to ASCVD (34–36).

Anthracyclines have been shown to impact ABCA1 and cholesterol efflux perhaps by interfering with the activity of the upstream regulators LXR-alpha and PPAR-gamma (37). The mechanism of anthracycline-induced atherosclerosis pathways was investigated by Sharma et al. in their study of the *in vivo* effects of doxorubicin, epirubicin, and other agents on human hepatocytes, isolating the effect of each chemotherapy agent on genes involved in lipid metabolism (38). Their study showed that doxorubicin was associated with decreased mRNA transcription of ABCA1. Overall impairment by doxorubicin resulted in a dose-dependent 20–30% cholesterol efflux in their cell model. A similar effect was seen with epirubicin (38). In a study of doxorubicin-treated myocytes and a mouse model, doxorubicin-treated cells exhibited higher cholesterol and cholesterol precursor levels (39). Sharma et al. also showed that anthracyclines reduced HMGCR activity, which in theory should reduce LDL levels (38). Others have shown a significant increase in apolipoprotein B, a known risk factor for ASCVD, with anthracycline use (37).

Clinical studies by Lu et al. (34) and He et al. (36) showed a consistent association of increased total cholesterol (TC), LDL, and triglycerides (TG) measured at the conclusion of anthracycline-predominant combination chemotherapy in a combined sample of over 1,100 patients. Anthracycline effect on HDL was less consistent and was associated with decreased levels in the former study, in line with the decrease in ABCA1 reported in the above mechanistic studies. Limited long-term studies have evaluated lipid levels after anthracycline therapy in isolation, so the duration of this effect is less clear. However, in a group of 433 patients with early breast cancer, Arpino et al. (40) showed anthracycline therapy, in combination with cyclophosphamide followed by taxane therapy, was associated with elevations in TC, LDL, and TG at 24 months of follow up. This provides some evidence that the duration of anthracycline effect on lipid metabolism is durable. Overall, multiple lines of evidence across basic science and clinical studies suggest that anthracyclines may play a role in promoting dyslipidemia, but further study is required.

#### Taxanes

Taxanes act primarily by stabilizing GDP-bound tubulin in microtubules, preventing effective function of microtubules for cell division (41). They are versatile drugs used to treat various solid tumors, and are a core component of breast cancer therapy (41). In a study of 80 women with breast cancer, a single infusion of paclitaxel was shown to effect expression of at least 188 proteins (42). Proteomic analysis showed that many of these proteins were crucial in lipid metabolism, especially those that involve lipoproteins. Sharma et al. (38) demonstrated increased HMGCR activity in human hepatocytes induced by paclitaxel. Additionally, reduced apolipoprotein-B activity and LDL receptor expression (important for clearance of LDL from the blood stream) were noted with paclitaxel.

Because taxanes are used almost exclusively as combination therapy (often with anthracyclines), it is difficult to assess their unique impact on *in vivo* lipid metabolism. In a sub-group analysis of patients who received regimens with and without taxane therapy, He et al. (36) showed that taxane-containing regimens resulted in higher magnitude of dyslipidemia than non-taxane containing regimens. Other studies similarly suggest that taxane-inclusive regimens are associated with dyslipidemia especially when used in combination with anthracyclines (34, 35, 40).

#### Tyrosine kinase inhibitors

Tyrosine kinase inhibitors (TKIs) are a mainstay of therapy for gastrointestinal, genitourinary, hematologic, and lung cancers. They are a diverse class of agents targeting various kinases involved in pathways related to tumor angiogenesis. The off-target effects of TKIs can impact lipid metabolism or promote cardiotoxic side effects. Imatinib, a first-generation TKI targeting BCR-ABL1, reduces cytoplasmic phosphorylation of LDL receptor related protein, a key component of LDL signaling that is involved in activating lysosomal enzymes, glucose-induced insulin secretion, and cholesterol metabolism. For example, in a study of rabbits fed a high cholesterol diet, imatinib therapy reduced cholesterol levels and the toxic effects produced by hyperlipidemia in the aorta and liver. These effects were mediated *via* significant decreases in lipid levels, C-reactive protein, and hepatic and other enzymes, suggesting that multiple pathways are involved in the atherosclerotic and vasculo-toxic effects of imatinib (43). Other TKIs that act on PDGF-R may have a similar effect (44), while TKIs that act *via* different target receptors may have opposing or synergistic effects.

##### Hematologic cancers

TKIs are a key therapeutic agent in chronic myelogenous leukemia (CML) (45). Since imatinib revolutionized CML therapy, multiple other TKIs targeting various receptor pathways have entered the market (45). Second generation agents include nilotinib, dasatinib, and bosutinib. Ponatinib is a third-generation agent used in cases of treatment failure (45). Though the mechanisms through which TKI therapies alter cardiovascular physiology are poorly understood, clinical data suggests numerous cardiotoxic side effects.

As a class, BCR-ABL1 TKIs have been found to have varying toxic effects, including adverse events that may be lipid-independent, such as vascular toxicity, cardiotoxicity, and pulmonary hypertension (45). Among vascular toxicities, peripheral arterial disease, cerebral vascular accidents, ischemic heart disease, hypertension, and hyperglycemia are a few of the better-known adverse effects (46, 47). The first-generation agent imatinib likely has a protective effect on lipid metabolism. In a case series of 9 patients with hyperlipidemia and CML, 8 of 9 patients experienced normalization of lipid levels within 30 days of starting imatinib without the use of cholesterol-lowering agents (44). In a study of 40 patients with CML, of whom 19 were on statin therapy concurrently, after 3 months of imatinib treatment TC, TG, LDL, and non-HDL cholesterol decreased while HDL increased. The lipid-lowering effect of imatinib was similar between the statin and non-statin groups (48). Further research is needed to identify the intermediaries through which this effect occurs, as well as its clinical implications considering long-term use of lipid-lowering agents.

Studies to date on TKI therapy suggest that imatinib may have an atheroprotective effect, while other TKIs may have a more deleterious effect. Ponatinib targets a gatekeeper mutation in the BCR-ABL protein, T3151, that confers resistance to other TKIs among CML patients, but after approval was noted to be linked to myocardial infarction or stroke in 24–48% of patients (49). One study using mouse models suggests that imatinib and ponatinib decrease plasma cholesterol levels and that imatinib can increase plaque stability. The same study found that ponatinib and nilotinib may increase mRNA expression of coagulation factors that activate the clotting cascade, thus inducing a prothrombotic state that may be the driver of increased vascular adverse events (50).

Compared to imatinib, nilotinib, and dasatinib have shown greater affinity for the BCR-ABL1 tyrosine kinase protein and thus more effective BCR-ABL1 inhibition, largely replacing imatinib as first line therapy for CML (51). In small studies available to date, nilotinib as first or second line therapy for CML was associated with increased TC, LDL, and HDL, higher risk of peripheral arterial disease, and worsened glycemic profile, suggesting lipid-dependent and lipid-independent toxicity (52–54). In another study of 57 patients with CML treated with either imatinib or nilotinib, imatinib was associated with a significant increase in HDL and decrease in TG and a non-significant decrease in TC and LDL from baseline compared to 6 month follow up. Nilotinib was associated with a significant increase in TC, LDL, and HDL and a decrease in TG, similar to prior studies (55). Overall, among agents active against BCR-ABL, it appears nilotinib increases risk of dyslipidemia, imatinib may decrease risk, and risk with other agents requires further study. The mechanism for these disparate effects among similar agents is not yet understood.

##### Solid tumors

Lipid-modulating effects of the more novel TKIs used in gastrointestinal, genitourinary, and lung malignancies are still being elucidated. In a study of 299 patients with lung cancer, patients treated with gefitinib (an EGFR TKI) had lower TC levels after 30 days of treatment (56). Lorlatinib, a third generation TKI used in ALK-positive non-small cell lung cancer, was found to induce hypercholesterolemia in 82.4% of patients and hypertriglyceridemia in 60.7% of patients. The mechanism of this effect is not known; however, one hypothesis is that lorlatinib has off-target effects on tropomyosin receptor kinase B (TrK-B), which is involved in cholesterol recognition (57, 58). Sunitinib, a novel TKI that acts on FGF, VEGF, and PDGF-R, was found to increase TC, LDL, HDL, non-HDL cholesterol, and TG in a study of 127 patients with metastatic renal cell carcinoma, of which 79 were receiving the drug. Paradoxically, higher increase in serum lipids, particularly in all 5 parameters, was associated with better progression-free survival and overall survival in this study (59). Additional studies to confirm this paradoxical relationship between increased lipid levels and better overall survival are warranted, and at the very least suggest that lipid metabolism is an important factor but not the only one in determining mortality risk in TKI recipients.

#### Mechanistic target of rapamycin inhibitors

The mechanistic Target of Rapamycin (mTOR) signaling pathway is involved in regulation of gene transcription and protein synthesis related to cell proliferation and immune cell differentiation (60). Primarily used as anti-rejection agents, mTOR inhibitors such as everolimus and temsirolimus are sometimes used as targeted anti-neoplastic therapy. mTOR inhibitors increase TG and LDL levels by reduced expression of lipogenic enzymes and LDL receptors, respectively (61). Kasiske et al. reviewed 17 randomized control trials of mTOR inhibitors and found 14 trials with an increase in TC and/or TG compared to the non-mTOR comparator groups (62). Additionally, use of lipid lowering therapy was more common in the mTOR groups. However, all trials evaluated mTOR inhibitor use in kidney transplant recipients, in which the doses of mTOR inhibitors were lower than that for anti-neoplastic treatment. In a phase I clinical trial of the mTOR inhibitor deforolimus for treatment of advanced malignancies, TC exhibited a dose response relationship of 7.4 mg/dL per 10 mg increase in dose (maximum dose 75 mg) (63). In a phase III trial of temsiromilus for treatment of metastatic renal-cell carcinoma, hypercholesterolemia was seen in 24% of patients compared to 4% in the interferon alfa comparator group (64). However, the clinical significance of mTOR inhibitor induced dyslipidemia remains unclear given that mTOR inhibition may actually inhibit pathways involved in the formation of atherosclerosis (65). The long-term impact of mTOR inhibition on cardiovascular risk is unclear, as findings of reduced cardiovascular risk in patients on mTOR inhibitors are confounded by more frequent use of lipid lowering therapy in response to dyslipidemia (65, 66). Further study is needed evaluate the effect mTOR inhibitors on atherosclerotic disease.

#### Alkylating agents

Alkylating agents cross-link strands of DNA and RNA to prevent cell division (67). Fulminant cardiotoxicity is a rare side effect of high-dose cyclophosphamide, the most common alkylating agent (68). However, the association between alkylating agents and dyslipidemia is unclear. Sharma et al. showed *in vitro* exposure of human hepatocytes to cyclophosphamide did not affect metabolic pathways involved in lipogenesis (38). In animal models, cyclophosphamide was shown to induce dyslipidemia in rats at toxic doses (69). In contrast, treatment with low dose cyclophosphamide was associated with reduced progression of atherosclerotic disease in mice (70). Clinical studies in humans show cyclophosphamide regimens that do not include anthracyclines do not affect lipid profiles and may even be associated with improved LDL (38). Data on dyslipidemia associated with other alkylating agents is lacking, likely due to limited usage.

#### Platinums

Platinum complexes are positively charged platinum ions surrounded by negatively charged anions that cross-link DNA to inhibit transcription, resulting in failed mRNA translation and ultimately promoting cell death (71). While cholesterol metabolism appears to affect the efficacy of platinum agents (72, 73), there are minimal human studies linking platinum agents to long-term changes in cholesterol levels (74). Najam et al. investigated the effect of the platinum drugs cisplatin and oxaliplatin on rats. They noted increased levels of LDL and TG 30 days after cessation of therapy compared to controls injected with normal saline (75). Histologic review of sacrificed rats showed evidence of myofibrillar loss and vessel wall thickening in those treated with cisplatin but not oxaliplatin, but these results have not been replicated in human studies. In clinical studies in humans, an adjuvant combination regimen primarily composed of cisplatin, carboplatin, and nedaplatin was found to be associated with increased levels of TC, LDL, HDL, and TG at the end of the treatment period (76). However, in two other studies in human subjects, no changes in plasma cholesterol were seen shortly after cisplatin therapy (77) nor after >5 year follow up (74). Overall, there is limited evidence to suggest treatment with platinum agents leads to dyslipidemia.

#### Anti-metabolites

Anti-metabolites are purine and pyrimidine analogs that get incorporated into DNA to interfere with cell proliferation (78). They are a very diverse group of medications used to treat various malignancies (79). 5-Fluorouracil (5-FU) was associated with cholesterol lowering in a study in rabbits, but this link has not yet been established in humans (80). Methotrexate may cause decreases in cholesterol levels through alterations in expression of ABCA1 and 27-hydroxylase enzymes (81), an effect seen in a study of rheumatoid arthritis patients taking the drug (82). However, in the Cardiovascular Inflammation Reduction Trial, low dose methotrexate compared to placebo minimally reduced LDL, TG, and HDL but no cardiovascular benefit was observed (83). In patients with colorectal cancer treated with a predominantly anti-metabolite regimen of 5-FU and capecitabine, TC, HDL, and TG increased while LDL decreased at the end of the treatment period (84). Capecitabine alone has been rarely associated with extremely elevated TG levels (85). Other anti-metabolites have no known association with dyslipidemia in the literature and the overall impact of the drug class on ASCVD risk is still unclear.

#### Hormone therapy

Estrogen and testosterone affect several regulators of cholesterol expression and transport (86, 87). For example, liver HMGCR is inhibited by estrogen, thereby resulting in decreased cholesterol synthesis (88). Additionally, testosterone deficiency has been shown to result in decreased expression of the nuclear receptors LXR and PPAR-gamma which are crucial in controlling the level of serum cholesterol (89). The inhibition of sex hormones, mainly estrogen and testosterone, are a mainstay of breast and prostate cancer therapy (90). Hormone therapy agents act agonistically or antagonistically in different tissues, thus their effects on cholesterol homeostasis are variable. Given that sex-specific hormones are closely involved in cholesterol metabolism, deficiencies in estrogen (91, 92) and testosterone (93–95) are associated with worsening lipid profiles.

In patients with breast cancer, treatment with selective estrogen receptor modulators (SERMs) such as tamoxifen have a protective effect against ASCVD and dyslipidemia (96). SERMs act as estrogen antagonists at treatment target tissues such as the breast or uterus, however, in most other tissues they mimic the effect of estrogen (97). Thus, they can provide a protective effect against chemotherapy-induced dyslipidemia (98). In a study of 55 breast cancer patients in India, participants received 20 mg daily of adjuvant tamoxifen after breast cancer surgery and had significant reductions in TC and LDL (99). However, a separate study of 141 breast cancer patients receiving 3 years of SERM therapy showed that alterations in the lipid profile normalized within 6 months of drug discontinuation (100). Elevations in TG after SERM treatment have occurred in some studies (101, 102) but not others (99, 103).

In a metanalysis of RCTs with variable follow up intervals, tamoxifen reduced cardiovascular events by 25–35% compared to placebo (104). In large scale study of 3,449 breast cancer patients who completed 2 or 5 years of tamoxifen therapy, women 50–59 years of age in the 5-year therapy arm experienced 35% reduction in cardiovascular events and 59% reduction in cardiovascular mortality at 10-year follow up vs. placebo. However, this effect was not found in other age groups (105). In addition, when prophylactic tamoxifen was studied in patients with and without cardiovascular disease at risk of developing breast cancer, there was no change in cardiovascular event rates associated with tamoxifen use (106). Finally, tamoxifen is associated with increased risk of venous thromboembolism and pulmonary embolism, though these end points may have been excluded in studies evaluating cardioembolic events (107).

Aromatase inhibitors (AI) reduce circulating estrogen by reducing production of the hormone (108). Compared to SERMs they are associated with increased cardiovascular risk (104). In a study of over 17,000 patients in the United Kingdom newly diagnosed with breast cancer and initiated on either an AI or tamoxifen, initial treatment with an AI was associated with a risk factor adjusted hazard ratio of 1.5 compared to those initiated on tamoxifen. In one systematic review and meta-analysis, extended treatment (>5 years after initial diagnosis) with AIs was associated with 1.18 higher odds of cardiovascular events compared to placebo (109). Additional data on cardiovascular outcomes comparing AI vs. placebo is lacking and thus comparison of cardiovascular outcomes against protective SERMs may make AIs appear more harmful. However, AIs do appear to increase dyslipidemia at least 3 months after cessation of therapy, though the full duration of effect is unknown (110). Sequential therapy with SERMs prior to AIs may mitigate this effect (111).

Androgen deprivation therapy (ADT) using hormonal treatment such as gonadotropin-releasing hormone antagonism, or less commonly, bilateral orchiectomy, is a standard component of treatment of advanced prostate cancer (112). The typical lipid profile change has been found to be an increase in TC, TG, LDL, and HDL (113). Additionally, increased arterial stiffness at 6 months has been reported following ADT and correlates with increasing cholesterol levels post-therapy (114). Increases in insulin resistance and incidence of type II diabetes have also been reported after ADT. Risk for cardiovascular disease has been shown to increase 20% after at least 1 year of ADT (115) and long-term risk may be increased with chemical ADT vs. orchiectomy (116). However, data translating this risk to higher cardiovascular mortality is mixed (113).

#### Chemotherapy induced ovarian failure

Due to high cell growth rates, ovarian follicles are exquisitely sensitive to chemotherapy (117). Incidences of chemotherapy-induced ovarian failure (CIOF) as high as nearly 80% have been reported in pre-menopausal cancer patients (118). A review article by Roeters van Lennep et al. summarizes these effects. Chemotherapy can induce apoptosis of ovarian follicles, damage to follicular blood supply, and accelerated maturation of follicles that ultimately lead to premature ovarian failure. Due to the subsequent lack of estrogen production, premature ovarian failure is an established risk factor for ASCVD that may be independent of lipid homeostasis (119). Studies among women with premature ovarian failure suggest this risk may be due to increased abdominal fat, elevated inflammatory markers, or increased TG without a clear link to total cholesterol or LDL levels (120, 121). However, in a study of patients on cyclophosphamide, methotrexate and 5-FU with premature ovarian failure, risk of dyslipidemia was significantly increased (122). Chemotherapy regimens with a high risk for ovarian failure include those with cyclophosphamide, methotrexate, anthracyclines, and 5-FU for 6 or more cycles, especially in patients age > 40 years (123). SERMs may offer protection against the dyslipidemia seen in CIOF. In a study of breast cancer patients treated with a majority cyclophosphamide, methotrexate, and 5-FU regimen in addition to tamoxifen vs. control, 53% of patients had ovarian failure (124). Those treated with tamoxifen after chemotherapy had improved lipid profiles at 12 months after tamoxifen initiation, while those that received placebo after chemotherapy had no change in their dyslipidemia (124). The most effective methods for prevention and treatment of CIOF are the subject of ongoing research (125).

### Non-chemotherapy cancer treatments

Cancer treatment in the recent era is most often multimodality, and non-chemotherapy treatment agents affect dyslipidemia and ASCVD risk. Radiation therapy, often used in conjunction with chemotherapy therapy regimens, is an independent risk factor in the development of ASCVD (126). Explosive growth in the use of immunotherapy and stem cell transplant has changed the typical treatment regimens for many common cancer types. These modalities have been associated with increased ASCVD risk but association with dyslipidemia is less clear.

#### Immunotherapy

Studies of the effects of immunotherapy on lipid metabolism are limited. Chimeric antigen receptor-T cells and Bispecific T cell Engager therapy are growing areas of immunotherapy, particularly for hematologic malignancies, but their effects on lipid metabolism are unknown. Immune checkpoint inhibitors (ICIs) are an increasingly common therapy for a variety of cancers and known to have multiple cardiometabolic toxicities (127, 128). ICIs are monoclonal antibody antagonists to CTLA-4, PD-1, and PD-L1 (129). Abnormal PD-1 and PD1-L expression, as occurs with ICI use, can lead to progression of atherosclerosis (129). Alterations in T-cell mediated intraplaque immune responses are thought to make atherosclerotic disease more vulnerable to rupture. ICI inhibition of key regulatory pathways in cardiomyocytes is thought to promote myocarditis, vasculitis, atherosclerosis, arrhythmias, and pericardial disease. A recent study of 2,842 patients with a variety of cancers undergoing ICI therapy found that those who received ICIs had 3-fold higher risk of experiencing atherosclerosis-mediated cardiac events (130). In a case-control analysis of the same population, the observed rate of cardiovascular events in the 2 years after ICI therapy was 6.55 per 100 person-years compared to 1.37 in the 2 years prior to therapy. In an imaging subgroup analysis of 40 patients, atherosclerotic plaque volume was 3 times higher with ICI therapy (130). A key question remains whether clinicians should be more aggressive with treating CVD risk factors given this population's higher propensity for adverse cardiac events. Moreover, there seems to be evidence that ICIs can enhance ASCVD risk, but their specific effects on lipid metabolism remain to be elucidated.

#### Stem cell transplant

Hematopoetic stem cell transplant (SCT) patients are at elevated risk of hypertension and diabetes, and further evidence suggests that SCT is an established accelerator of atherosclerosis (131). A retrospective analysis of 194 patients who underwent allogeneic SCT and survived more than 100 days found that 42.8% of patients developed hypercholesterolemia and 50.8% of patients developed hypertriglyceridemia. The development of chronic graft-vs. host disease (GVHD) and use of steroids were associated with development of hypercholesterolemia, while use of calcineurin inhibitors was not (132). Another retrospective analysis of 761 patients who underwent allogeneic SCT and survived more than 100 days found an incidence of hypercholesterolemia and hypertriglyceridemia of 73.4% and 72.5%, respectively, at 2 years post-transplant (133). This study also found an association between acute GVHD and hypercholesterolemia and hypertriglyceridemia, and the authors proposed a role for GVHD-related liver dysfunction in the role of lipid dysregulation. Of note, statin use in this population effectively lowered TC, TG, and LDL levels (133). Thus, as SCT patients continue to experience better long-term survival, SCT must be recognized as a risk factor for accelerated atherosclerotic disease to guide appropriate prophylaxis and treatment (134).

#### Radiation therapy

Localized radiation therapy (RT) has been shown to promote the development of atherosclerosis in affected tissues (25). Radiation therapy causes local endothelial damage which prompts an inflammatory cascade resulting in atherosclerosis (25). Inflammation and subsequent host immune and healing response lead to fibrosis, stenosis, and development of early atherosclerotic disease. Dyslipidemia has not been identified as a major contributor to early ASCVD in patients treated with RT and there is little mechanistic data in basic science studies to suggest it may play a significant role. In an *in vitro* study, human bronchial epithelial cells were exposed to radiation doses known to cause cell dysfunction in humans (135). Increases in cholesterol pathway enzymes and a 10–30% increase in intracellular cholesterol were detected 7 days after RT. However, whether this outcome would translate to *in vivo* studies in a meaningful way is unknown. Small studies in breast (136) and prostate (137) cancer patients showed an association of RT with reduced dyslipidemia. Large-scale clinical trials regarding dyslipidemia after RT—along with treatment strategies—are needed.

## Clonal hematopoiesis of indeterminate potential

### CHIP mutation predisposition to ASCVD independent of dyslipidemia

The identification of effects of CHIP adds a new dimension to the interweaving of the cardiovascular and cancer biology spheres. CHIP mutations create a hematologic pre-malignant state that can progress to hematologic malignancies such as leukemia, lymphoma, and myeloma at a rate of 0.5–1% per year (11). However, even in CHIP carriers who do not progress to hematologic malignancy, the all-cause mortality rate may be 40% higher and incidence of coronary heart disease twice as high as those without CHIP (11, 138). Thus, an emerging body of literature seeks to characterize the risk factors associated with CHIP development and its key behaviors. To date, CHIP mutations do not appear to increase risk of dyslipidemia. In fact, increases in ASCVD risk with CHIP mutations have been shown to be independent of traditional cardiovascular risk factors such as dyslipidemia, type 2 diabetes, hypertension, and smoking status (139). One study notes that human carriers of a CHIP mutation, particularly DNMT3A, TET2, ASXL1, and JAK2, had up to 1.9 times the risk of coronary heart disease and 4 times the risk of myocardial infarction compared to non-carriers. The same study evaluated the effects of CHIP mutations on atherosclerosis in mice and found that mice engrafted with bone marrow obtained from TET2 knockout donors had larger atherosclerotic lesions in the aorta and aortic root than mice engrafted with control bone marrow (139). In a retrospective cohort study, patients with CHIP mutations that underwent treatment for AML had a 1.7 times higher incidence of ASCVD events than those without mutations (140). In a study of over 35,000 people without history of coronary vascular disease in the U.K. Biobank, those with DNMT3A or TET2 mutation had 27% higher risk of CVD in 6.9 years of follow up than those without the mutations (141). JAK2 mutations have been strongly linked to arterial thrombosis, conferring a 2-fold increase in events in multiple studies according to a recent review article by Leiva et al. (142). Additionally, CHIP mutations appear to exhibit a dose-response relationship with ASCVD, with the presence of more CHIP mutations increasing cardiovascular risk (139, 142). Thus, the association of CHIP with dyslipidemia and ASCVD in cancer patients, and potential mechanistic links between the two, requires ongoing study.

## Discussion

### Lipid-lowering in cancer patients

The effect of cancer and cancer therapy on atherosclerosis suggests that cancer patients are at higher risk of ASCVD-related morbidity and mortality, necessitating greater attention on prophylaxis and treatment of cardiovascular disease in this population. A recent review article by Zullig et al. summarizes the evidence regarding pharmacologic and non-pharmacologic management of cardiometabolic comorbidities in cancer survivors (143). Statins, competitive inhibitors of HMGCR, and proprotein convertase subtilisin/kexin type 9 serine protease (PCSK9) inhibitors are the mainstays of preventive therapy in cancer patients as in the general population (144, 145). A fundamental challenge facing cardiooncologists and lipidologists is the approach to treatment and prevention of dyslipidemia in this vulnerable population. Current practice advises treatment of dyslipidemia in cancer patients using the same guidelines as patients without cancer, but the response to lipid-lowering therapy in these patients is yet to be studied in large-scale trials.

Statins may benefit oncology patients through their cholesterol-lowering effects as well as an anti-cancer effect. For example, the use of statins prior to cancer diagnosis has been linked to reduced risk of developing certain cancers such as pancreatic cancer and non-Hodgkin lymphoma (146–148). Additionally, statins may enhance the anti-tumor properties of some chemotherapeutic agents. For example, in two studies of patients with metastatic renal cell carcinoma receiving anti-VEGF or mTOR-inhibitors and lung cancer receiving EGFR-TKI therapy, patients treated with statins concurrently had improved overall survival compared to statin non-users (149, 150). Similarly, a randomized placebo-controlled trial of 89 participants found that prophylactic use of rosuvastatin at the time of anthracycline therapy prevented reductions in ejection fraction, left atrial diameter, and E/e' ratio (151). Finally, three large meta-analyses of patients using statins did not show an association between statin use and increased risk of developing cancer (152, 153). Though the mechanisms by which statins promote an anti-cancer effect are not well-known, this area warrants further research given that traditional thresholds of ASCVD risk may exclude oncology patients that would benefit from preventative therapy.

PCSK-9 inhibitors are an established alternative or adjunct therapy to statins for cholesterol reduction. They have profound LDL-lowering effects that have been shown to reduce cardiovascular events (154, 155). The use of PCSK-9 inhibitors in patients with cancer is less well-studied, though early basic science evidence suggests that their cholesterol-related and non-cholesterol-related properties may have consequences for cancer as well (156, 157). For example, one study found that PCSK-9 inhibition in mice potentiated the anti-tumor effects of ICI therapy, although the direct effects of lipid metabolism have yet to be studied (158).

Non-pharmacologic approaches to lipid-lowering in cancer patients are also important. Exercise oncology is a growing area of interest to promote health and longevity among cancer survivors (159). Tailored pre- and post-treatment exercise programs are being studied for their potential to reduce the detrimental effects of cancer therapy on the cardiovascular system as well as overall morbidity and mortality (159, 160). Among patients without cancer, exercise has been shown to increase blood lipid consumption and decrease lipid levels. Potential mechanisms include increased LPL activity, increased expression of ABCA1, increased plasma HDL formation, and increased LXR formation (161–165). While early studies suggest that exercise has cardiovascular benefit for cancer patients, a nuanced study of its effect on lipid metabolism in this population is yet to be conducted.

Thus, there is promising data to suggest that cancer patients with dyslipidemia may benefit from traditional treatment with statins or PCSK-9 inhibitors. The next frontier for clinicians and researchers is identification of more nuanced treatment guidelines for cardiooncology patients who may be at increased risk of atherosclerotic events. Further research into the changes in lipid metabolism attributable to various cancers and cytotoxic therapies is needed to inform such guidelines.

### Clinical guidance for managing dyslipidemia and ASCVD risk in cancer patients

Screening for ASCVD in the general population relies on traditional risk stratification tools, however, such tools do not often account for a history of cancer or cancer treatment. In adults who received cancer treatment as a child, the Childhood Cancer Survivor Cardiovascular Risk Calculator can estimate risk based on age, gender, and prior treatment (166). However, no such calculator exists for patients receiving cancer treatment as adults. Additionally, depending on baseline risk factors and cancer treatment course, risk of ASCVD in cancer survivors may be substantially underestimated. Formal clinical practice guidelines for prevention of dyslipidemia and ASCVD cancer patients are sparse due to limited data, but several societies advocate for more aggressive assessment of ASCVD risk in cancer patients (167–169).

While studies are underway to better characterize the increased ASCVD risk that cancer patients carry, clinicians treating the cancer patient should be aware of classes of cytotoxic therapy that are known to increase risk in long-term dyslipidemia. These include Anthracyclines, VEGF inhibitors, and ADT. Clinicians should consider more aggressive screening of cardiovascular risk factors in patients who have received or are receiving treatment with these agents. Additionally, clinicians should utilize the diagnostic tests used in cancer management to help risk stratify ASCVD risk. For example, coronary artery calcifications on chest imaging are associated with clinically significant ASCVD (170, 171). Moreover, baseline testing prior to anthracycline initiation, which includes ECG, echocardiogram, and occasionally cardiac magnetic resonance imaging, should be reviewed thoroughly for evidence of subclinical CAD (172–174).

Statin therapy should continue to be the mainstay of lipid lowering therapy in cancer patients and clinicians ought to consider lower thresholds of traditional ASCVD risk calculators to initiate or intensify therapy in cancer patients treated with chemotherapy associated with dyslipidemia or ASCVD, chest radiation, immunotherapy, or stem-cell therapy (Table 1). At this juncture, there is insufficient evidence to recommend PCSK-9 inhibitors as adjunct therapy for cancer survivors unless they have an indication specified in society guidelines, but further research is needed to explore specific indications for this medication in cancer survivors. A healthy lifestyle should be emphasized to cancer survivors to reduce risk of cancer recurrence and ASCVD (175, 176).

### Implication of cholesterol-related metabolites in cancer

Although the present review focuses on cytotoxic effects related to cholesterol, other related lipids have been implicated in cancer risk. Oxysterols are derivatives of cholesterol that act as direct activators of the nuclear receptor LXR (177). LXR activation can have direct anti-proliferative effects and inhibit cell cycle activation (178, 179). Others have shown that LXR agonism improves immune responses to immunotherapy (180). Thus, there is substantial interest in LXRs as potential targets for cancer (181). Complete loss of SREBP1 and SREBP2 through SREBP cleavage-activating protein deficiency inhibits cancer cell growth and viability through modulation of fatty acid desaturation activity, highlighting a key role for fatty acid regulation in cancer (182). Further study as to how cholesterol-related metabolites influence cancer development and therapeutic responses may have important implications for cardiovascular risk assessment in cancer patients and invite new approaches to cytotoxic drug development.

### Conclusion and future directions

Dyslipidemia is a crucial factor in the development of ASCVD. The increased incidence of ASCVD in cancer patients suggests the need for greater attention on prophylaxis and treatment of cardiovascular disease in this population, including study of potential precursors of both disease states, such as CHIP. Future research may include specific study of various cytotoxic therapies on incident ASCVD, as research to date suggests that these effects may be variable. Prospective studies are needed to assess dyslipidemia and the risk of ASCVD after each class and combination of chemotherapy treatment to better predict the likelihood of an ASCVD event in cancer survivors (183). Moreover, there are multiple lipid-independent effects of cancer therapy on ASCVD, such as vascular toxicity and CIOF, that must be studied in conjunction with the lipid-dependent effects outlined in this review for holistic understanding of the impact of cytotoxic therapy on ASCVD risk. Clinicians should be keenly aware of the unique impacts cytotoxic treatment regimens may have on short-term and long-term cholesterol metabolism. As cancer survivors live longer and the duration and variety of treatment regimens increases, the heterogenous relationships between dyslipidemia and accelerated ASCVD warrant close study. Thus, greater attention toward ASCVD risk in cancer patients is required, and early and aggressive efforts must be made to modify risk factors such as atherosclerosis. Importantly, research on treatment strategies with guideline-recommended lipid lowering therapies is critical to determine the most effective methods of reducing ASCVD risk. With better understanding of cancer therapy-related ASCVD risk, evidence-informed guidelines for screening and prevention can be implemented for this unique, varied, and vulnerable population.

## Author contributions

RB, ND, EY, and TS: envisioning, design, writing, and revision of the manuscript. All authors contributed to the article and approved the submitted version.

## Funding

TS receives research funding from the National Institutes of Health (Grant Nos. HL149766, HL139549, DK118086, and DK127232), American Heart Association, and Burroughs Welcome Fund outside of the present study. EY receives research funding from CSL Behring, Boehringer Ingelheim, and Eli Lilly outside of the present study.

## Conflict of interest

The authors declare that the research was conducted in the absence of any commercial or financial relationships that could be construed as a potential conflict of interest.

## Publisher's note

All claims expressed in this article are solely those of the authors and do not necessarily represent those of their affiliated organizations, or those of the publisher, the editors and the reviewers. Any product that may be evaluated in this article, or claim that may be made by its manufacturer, is not guaranteed or endorsed by the publisher.
