# Supplementary material for: Cancer therapy's impact on lipid metabolism: Mechanisms and future avenues
Source: Front Cardiovasc Med. 2022 Aug 9;9:925816. doi: 10.3389/fcvm.2022.925816 (PMC9396263; doi:10.3389/fcvm.2022.925816)
Supplement: Supplementary file 1 [file Table_1.docx]

Supplementary Table 1: Studies evaluating lipid profile changes after chemotherapy.

| Agent Class | Predominant Agent | First Author | Year | Cancer Type | Patients | Follow up (mo) | TC | LDL | HDL | TG |
| --- | --- | --- | --- | --- | --- | --- | --- | --- | --- | --- |
| Anthracycline | N/a | Tian | 2019 | Breast | 69 | 6 | **-** |  | **-** | **-** |
|  | Epirubicin | Lu | 2020 | Breast | 683 | 0 |  |  |  |  |
|  | Epirubicin | He | 2020 | Breast | 500 | 0 |  |  |  |  |
| Taxane | N/a | Tian | 2019 | Breast | 199 | 6 |  |  | **-** |  |
|  | Docetaxel, paclitaxel | Lu | 2020 | Breast | 139 | 0 | **-** | **-** |  |  |
|  | Paclitaxel | He | 2020 | Breast | 237 | 0 |  |  |  |  |
| TKI (Bcr-Abl) | Nilotinib | Rea | 2014 | CML | 27 | 12 |  |  |  |  |
|  | Imatinib | Ellis | 2017 | CML | 40 | 12 |  |  |  |  |
| TKI (VEGF) | Sunitinib  Axitinib  Sorafenib | Zhang | 2020 | RCC | 127 | 72 |  |  |  |  |
| mTOR Inhibitor | Deforolimus | Hartford | 2009 | Various | 46 | 1 |  | NR | NR | NR |
| Alkylating Agent | Cyclophosphamide | Li | 2018 | Breast | 394 | 0 |  |  |  |  |
| Platinum | Cisplatin | Koc | 2011 | Testicular | 39 | 110 | **-** |  | **-** | **-** |
|  | Cisplatin/carboplatin/nedaplatin | Luo | 2021 | NSCLC | 117 | 0 |  |  |  |  |
| Anti-metabolite | 5-FU, capecitabine | Wang | 2016 | Colorectal | 667 | 0 |  |  |  |  |
| ADT | Luprolide | Smith | 2002 | Prostate | 40 | 0 |  |  |  |  |
|  | Goserelin  Leuprorelin | Oka | 2016 | Prostate | 58 | 6 |  |  |  | NR |
| AI | Anastrazole | Anan | 2011 | Breast | 69 | 36 | **-** | **-** | **-** | **-** |
|  | Exemestane  Letrozole | Bell | 2013 | Breast | 95 | 3 |  | **-** |  | **-** |
| SERM | Tamoxifene | Love | 1994 | Breast | 70 | 60 |  |  | **-** | **-** |
|  | Toremefine | Anan | 2011 | Breast | 69 | 24 |  |  |  |  |

Increased

Decreased

**-** No change

NR: Not reported

Abbreviations: ADT, androgen deprivation therapy; AI, aromatase inhibitor; HDL, high-density lipoprotein; LDL, low-density lipoprotein, mTOR, mammalian target of rapamycin; SERM, selective estrogen receptor modulator; TC, total cholesterol; TG, triglyceride; TKI, tyrosine kinase inhibitor; VEGF, vascular endothelial growth factor.
